# Bradykinin B1 receptor signaling triggers complement activation on endothelial cells

**DOI:** 10.3389/fimmu.2025.1527065

**Published:** 2025-02-07

**Authors:** Ingrid Lopatko Fagerström, Alexandra Gerogianni, Markus Wendler, Ida Arvidsson, Ashmita Tontanahal, Ann-Charlotte Kristoffersson, Fatimunnisa Qadri, Michael Bader, Diana Karpman

**Affiliations:** ^1^ Department of Pediatrics, Clinical Sciences Lund, Lund University, Lund, Sweden; ^2^ Max Delbrück Center for Molecular Medicine in the Helmholtz Association, Berlin, Germany; ^3^ German Center for Cardiovascular Research (DZHK) Partner Site Berlin, Berlin, Germany; ^4^ Charité – Universitätsmedizin Berlin, Berlin, Germany; ^5^ Experimental and Clinical Research Center, a cooperation between the Max-Delbrück Center for Molecular Medicine in the Helmholtz Association and the Charité -Universitätsmedizin Berlin, Berlin, Germany; ^6^ Institute of Biology, University of Lübeck, Lübeck, Germany

**Keywords:** bradykinin, complement, kallikrein–kinin system, glomerular endothelial cells, kidney, mice

## Abstract

**Introduction:**

The complement and kallikrein-kinin systems (KKS) are both activated during vascular inflammation, and there are many known interactions between the two systems. This study investigated if KKS activation induced complement activation on endothelial cells, and if activation was dependent on bradykinin B1 receptor (B1R) signaling.

**Methods:**

KKS was activated in normal human serum by kaolin or activated factor XII (FXIIa). ADP-preactivated primary glomerular endothelial cells (PGECs) were incubated with serum, with or without kaolin or FXIIa, and with or without the B1R antagonist (R715) or the inositol triphosphate receptor (IP3R) inhibitor 2-aminoethoxydiphenyl borate (2-APB). Complement factors C3a, factor Ba and C5b-9 were evaluated by ELISA or immunoblotting. B1/B2 receptor double knock-out and wild-type mice were injected with lipopolysaccharide from *E. coli* B5:O55, to induce KKS activation.

**Results:**

Supernatants from PGECs incubated with serum exposed to kaolin or FXIIa exhibited higher levels of Ba and C5b-9, which were significantly reduced in the presence of the B1R antagonist. Complement activation induced by FXIIa was also reduced in the presence of the IP3R inhibitor. Likewise, cell lysates showed higher levels of C3a and C5b-9 in the presence of kaolin and FXIIa, and complement activation was significantly reduced in the presence of the B1R antagonist. B1/B2 receptor double knock-out mice exhibited less C3 and C5b-9 deposition in glomeruli compared to wild-type mice.

**Conclusion:**

This study demonstrates that KKS activation contributes to complement activation on the endothelium by B1R signaling. Blocking the B1R may have a role in reducing complement deposition and its effects on the endothelium.

## Introduction

The kallikrein–kinin system (KKS) is activated during vascular inflammation such as vasculitis (1), in response to trauma (2), sepsis (3, 4), and myocardial infarction (5). In patients with hereditary angioedema, uncontrolled KKS activation and bradykinin (BK) release lead to vascular leakage and the development of angioedema (6). Activation of the KKS can occur by different routes, in a soluble or cell-bound manner. Circulating factor XII (FXII) is autoactivated to factor XIIa (FXIIa) on negatively charged surfaces (7), by lipopolysaccharide (LPS) (8, 9), collagen (10), or misfolded proteins (11) or artificially by kaolin (12). FXIIa activates prekallikrein to kallikrein, which in turn activates more FXII. An alternative pathway for FXII activation occurs on endothelial cells whereby FXII is autoactivated after binding to its complex receptor composed of cytokeratin 1 (CK1) and the globular head of the C1q receptor or urokinase plasminogen activator receptor (13). Prekallikrein bound to high molecular weight kininogen (HK) on endothelial cells is activated to kallikrein by prolylcarboxypeptidase (14). Kallikrein cleaves HK to release bradykinin, which is rapidly processed to des-Arg^9^-bradykinin (DABK) in plasma. BK binds to the constitutively expressed bradykinin B2 receptor (B2R) and DABK exerts its effects by binding to the inducible bradykinin B1 receptor (B1R). Expression of the B1R is induced during chronic inflammation.

B1R and B2R are G-protein coupled receptors (GPCRs), and both induce an increase in intracellular Ca^2+^ via the inositol 1,4,5 triphosphate(IP3)-receptor (15, 16). Ligand binding to kinin receptors activates the endothelial cell and causes conformational changes to the cell membrane (17), prostaglandin release (18), and nitric oxide production (19). In addition, B1R has been shown to induce neutrophil chemotaxis (20). These alterations to the cell and its surroundings lead to increased vascular permeability, vasodilation, and an inflammatory response.

The complement system is also activated during vascular inflammation. All three pathways of complement activation, the classical, lectin, or the alternative pathway, lead to the formation of a C3 convertase, which cleaves C3 into the opsonin C3b and the anaphylatoxin C3a. C3b forms the C3 convertase with factor Bb and can bind to formed C3 convertase, thus generating the C5 convertase. This initiates the terminal pathway whereby C5 convertase cleaves C5 to yield C5a and C5b. C5b forms a complex with C6, C7, C8 and multiple C9s (21).The membrane attack complex (MAC or C5b-9) is formed and can lead to pathogen elimination, or, in sublytical concentrations, to cell activation (22).

There are many known interactions between the complement system and the KKS. The KKS and the classical pathway of complement share the same inhibitor, C1-inhibitor (7). Moreover, kallikrein can cleave C3 and factor B (23, 24) and FXIIa can activate the classical pathway of complement by activating C1r and, to a lesser extent, C1s (25). Our group has shown that complement activation occurs on endothelial extracellular vesicles released from glomerular endothelial cells when vasculitis plasma was perfused over the cells. Complement deposition on the extracellular vesicles was reduced by B1R and B2R antagonists or by the C1 inhibitor, suggesting a possible interaction between the KKS and complement on glomerular endothelial vesicles. Furthermore, using a mouse model of glomerulonephritis, we reported that a B1R antagonist reduced glomerular C3 deposition (26).

The aim of the current study was to assess the interactions between the KKS and complement on glomerular endothelial cells and the intracellular pathway by which this activation occurs. The KKS was activated on glomerular endothelial cells by kaolin or FXIIa and cell supernatants and lysates assessed for complement C3a, Ba, and C5b-9. Experiments were performed in the presence of a B1R antagonist as well as an IP3 receptor inhibitor. KKS activation was induced *in vivo* in mice by LPS to assess glomerular C3 and C5b-9 deposition in wild-type as well as B1R/B2R double knock-out mice.

## Methods

### Serum samples

Whole blood was obtained from healthy adult controls without ongoing medications (n=6, one female) in vacutainer serum tubes (Becton Dickinson, Franklin Lakes, NJ, USA). Generally, the alternative pathway is more easily activated in samples from male donors (27). The tubes were left for 45 min at rt, allowing the blood to clot. The clot was removed by centrifugation at 1,500 × g for 10 min at 4°C, and the remaining serum was aliquoted and stored at −80°C until used. Serum samples were used in cell experiments. The study was conducted according to the Declaration of Helsinki with the approval of the Swedish Ethical Review Authority. Informed written consent was obtained from all participants.

### Primary glomerular endothelial cells

Primary glomerular endothelial cells (PGECs, Cell Systems, Kirkland, WA, USA) were used in all cell experiments and grown to confluency in a 12-well plate, as previously described (20). These cells express the kinin B1R (28). PGECs were preactivated by incubation with adenosine diphosphate (ADP, 1 mM, Sigma-Aldrich, St. Louis, MO, USA) for 30 min. Cells were washed and, in some experiments, preincubated with the B1R antagonist R715 (1 μM, Tocris Bioscience, Bristol, UK) or 2-aminoethoxydiphenyl borate (2-APB, 100 μM, Sigma-Aldrich, in dimethyl sulfoxide (DMSO)) to block the intracellular IP3 receptor, for 30 min at 37°C. Normal serum (1:4) was added with or without kaolin (0.1 mg/mL, Sigma-Aldrich) or with or without factor XIIa (FXIIa, Enzyme Research Laboratories, South Bend, IN, USA, 1.38 μg/mL for experiments with the B1R antagonist, 13.8 μg/mL for experiments with 2-APB) for an additional 30 min. The supernatant was removed, centrifuged at 10,000 × g for 2 min and stored at −80°C until analyzed. Cells were washed twice with PBS and lysed by adding radioimmunoprecipitation assay (RIPA) buffer (Santa Cruz Biotechnology, Dallas, TX, USA) followed by sequential freezing and thawing. Protein concentrations in lysates were assessed in using the Bradford assay kit (Bio-Rad Laboratories, Hercules, CA, USA). Cell lysates were stored at −20°C until analyzed.

### Kallikrein activity assay

**Figure 1:**
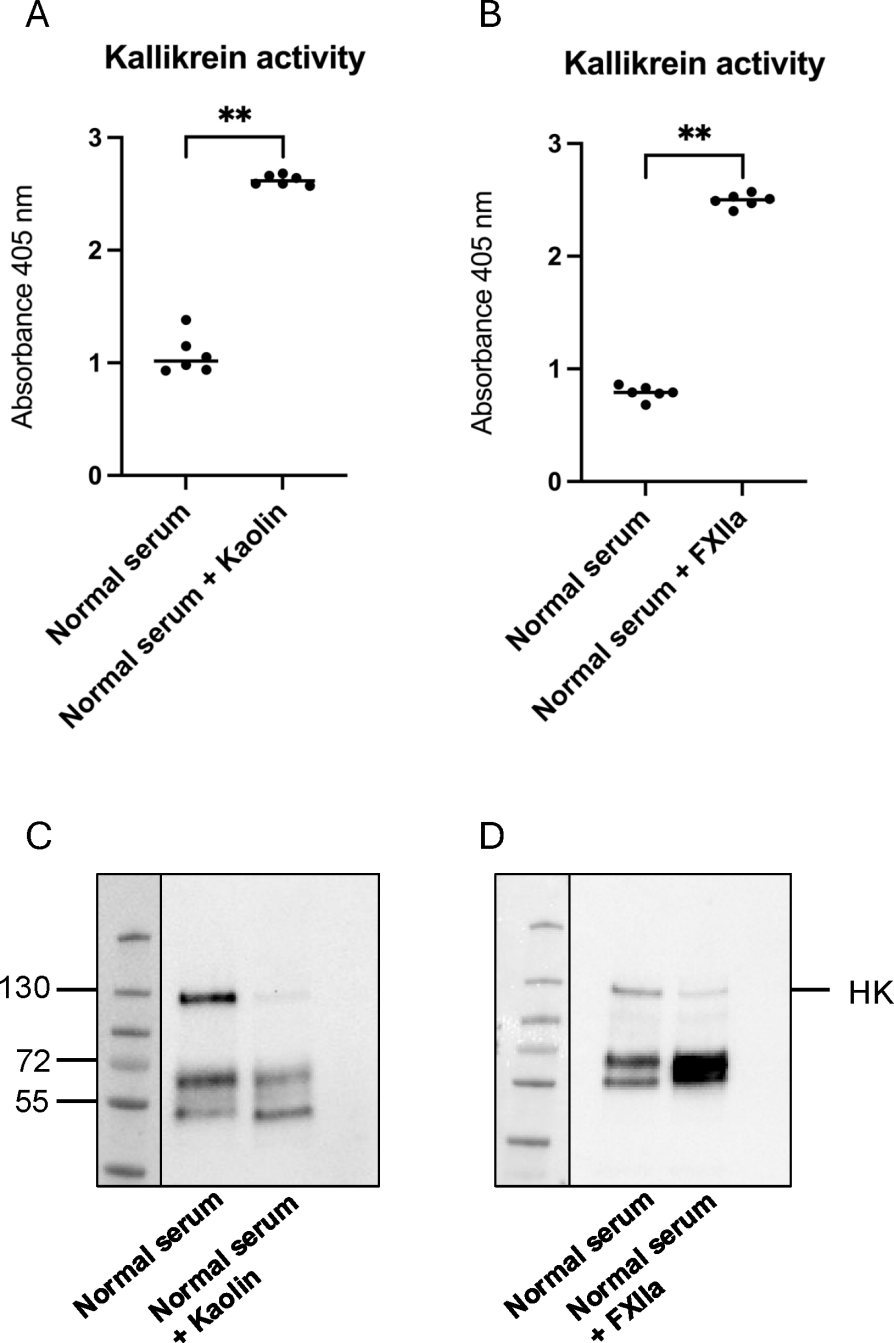
Activation of the kallikrein–kinin system in supernatants from cells treated with kaolin and FXIIa. **(A)** Kallikrein activity in supernatants from cells stimulated with kaolin (n=6) compared with controls (n=6). **(B)** Kallikrein activity in supernatants from cells stimulated with factor XIIa (FXIIa) (n=6) compared with controls (n=6). **(C)** Representative image of high molecular weight kininogen (HK) and its degradation products in supernatants from cells stimulated with kaolin (n=6). **(D)** Representative image of HK and its degradation products in supernatants from cells stimulated with FXIIa (n=6). **P <0.01.

Kallikrein activity in cell supernatants exposed to serum and stimulated with kaolin or FXIIa was analyzed using the chromogenic substrate PNAPEP 1902 (Cryopep, Montpellier, France). Samples were mixed 1:2 with PNAPEP 1902, and absorbance was measured at 405 nm at 60 min using a GloMax Discovery System (Promega, Madison, WI). Both kaolin (**Figure 1A**) and FXIIa (**Figure 1B**) were potent kallikrein activators, as expected. The B1R antagonist R715, in the presence or absence of kallikrein activators (**Supplementary Figures 1A, B**), and 2-APB in the presence of FXIIa, did not affect the assay (**Supplementary Figure 1C**).

### High molecular weight kininogen and C3a detected by immunoblotting

High molecular weight kininogen (HK) in cell supernatants and C3a in cell lysates were detected by immunoblotting. Samples were diluted 1:500 (HK) or to the same protein concentration (C3a) in sample buffer (0.14 M Tris buffer, pH 6.8 containing 4% (w/v) sodium dodecyl sulfate (SDS), 10% glycerol, (all from Sigma-Aldrich), 0.3% bromophenol blue (LKB Products, Bromma, Sweden)) with 10% 2-mercaptoethanol (Sigma-Aldrich) followed by boiling for 5 min. Samples were run on 4%–20% Mini-PROTEAN TGX gel (Bio-Rad Laboratories, Hercules, CA, USA) followed by transfer to a 0.2-μm polyvinylidene fluoride (PVDF) membrane (Bio-Rad Laboratories). Membranes were blocked with 10% casein (Vector Laboratories, Oxfordshire, UK) followed by 1-h incubation with sheep anti-human HK antiserum AS88 (1:5000) (29) or rabbit-anti human C3a (1:2000, Complement Technology, Tyler, TX, USA). After three washes in PBS-Tween (Medicago, Uppsala, Sweden), membranes were incubated with secondary antibody donkey anti-sheep horseradish peroxidase (HRP) (1:2,000, Dako, Glostrup, Denmark) or goat anti-rabbit HRP (1:1,000, Dako, Glostrup, Denmark) for 1 h at rt. After additional washing, the signal was detected by chemiluminescence using Pierce™ ECL Plus Western Blotting Substrate (Thermo Fisher Scientific, Waltham, MA, USA). Cell supernatants exhibited cleavage of HK induced by kaolin (**Figure 1C**) or by FXIIa (**Figure 1D**), as expected. The latter was not affected by the presence of the B1R antagonist R715 (**Supplementary Figure 2**). For C3a, the pixel intensity of the C3a band was measured and differences in C3a staining presented as fold change. Cells incubated with normal serum alone were defined as 1.

### C3a, Ba, and C5b-9 detection by ELISA

The presence of C3a, Ba, and C5b-9 in cell supernatants was analyzed by ELISA (Quidel Corporations, San Diego, CA, USA) according to the manufacturer’s instructions. Briefly, supernatants were diluted 1:200 (C3a), 1:2,000 (Ba), and 1:20 (C5b-9) in diluent media provided by the manufacturer and incubated for 1 h at rt. Sequential washing was performed, and conjugate was added to the wells and incubated for 1 h (C3a, Ba) or 30 min (C5b-9). Plates were washed five times and incubated with the substrate for 15 min, after which reactions were stopped and read at 450 nm. To analyze C5b-9 levels in cell lysates, samples were diluted to the same protein concentration and assessed as described above.

As a control C3 (24 μg/mL) and C5b-9 (1 μg/mL, both from Complement Technology) were incubated with FXIIa (1.38 μg/mL) for 30 min at 37°C in the absence of cells and serum and C3a as well as C5b-9 analyzed by ELISA, as described above. In addition, kaolin (0.1 mg/mL) and FXIIa (1.38 μg/mL) were incubated with normal serum for 30 min at 37°C in the absence of cells, and C3a detected by ELISA as described above. As kallikrein has been shown to cleave C3 in a pure system (23) and zymosan activates the complement system in serum (30), those were included as positive controls in experiments analyzing the presence of C3a.

### B1/B2 receptor knockout mice injected with lipopolysaccharide

C57BL/6N B1/B2 receptor knockout (31) and corresponding wild-type mice were bred in the animal facilities at Lund University. Female and male mice were used at 8–10 weeks of age. Double knockout mice were used as B1R-deficient mice overexpress the B2R (32). Mice were treated with 2 mg/kg lipopolysaccharide (LPS) from *Escherichia coli* B5:O55 (InvivoGen, San Diego, CA, USA) or PBS vehicle i.p. Mice were observed and sacrificed within 24 h when clinical signs of illness occurred, such as ruffled fur, decreased activity, hunched posture, squinting, or neurological defects (for the disease score, see **Supplementary Table 1**). All remaining mice were sacrificed at 24 h. Blood samples were taken while mice were under isoflurane anesthesia via heart puncture, into syringes filled with 100 μl of EDTA. Samples were centrifuged at 2,000 × g for 10 min and the plasma stored at −80°C until analyzed. Kidneys were fixed in 4% paraformaldehyde (PFA, Histolab Products AB, Askim, Sweden), embedded in paraffin and sectioned (4 μm) onto glass slides.

Animal experiments were approved by the regional Animal Ethics Committee (approval 17452-20) and performed in accordance with regulations of the Swedish Board of Agriculture and the European Directive on the protection of animals used for scientific purposes.

### Residual prekallikrein in murine plasma

Mouse plasma was analyzed using the kallikrein chromogenic assay described above. As kallikrein has a very short half-life in plasma, an alternative approach was applied measuring residual prekallikrein (33). To this end, plasma samples were diluted 1:4 in serum-free cell media (Lonza Group AG, Basel, Switzerland) and incubated with or without kaolin (0.1 mg/mL) for 10 min at 37°C. Samples were centrifuged at 10,000 × g for 2 min to discard kaolin. The supernatants were combined with PNAPEP 1:2, and absorbance was measured at 405 nm at 0 min and 60 min. Residual prekallikrein was calculated as the difference between samples incubated with kaolin at 60 min and the samples without kaolin at 0 min. A lower value indicates prekallikrein consumption in plasma.

### Urea levels in murine plasma

Urea was measured in EDTA plasma using a QuantiChrom Urea Assay kit (BioAssay Systems, Hayward, CA, USA) according to the manufacturer’s instructions. All samples were run as duplicates and detection carried out using the GloMax Discover System.

### Kidney tissues

Kidney sections were deparaffinized and stained with hematoxylin–eosin to evaluate histopathological changes. Tissues were visualized using a Nikon Eclipse Ti-E microscope with a Nikon color camera using NIS Elements AR software v.5.11.01 (Nikon Instruments Inc., Tokyo, Japan) and assessed in a blinded manner.

Immunofluorescence for C3 and C5b-9 was carried out as previously described (26). Sections were incubated with rabbit anti-C3 (4 μg/mL, Hycult Biotech, Uden, Netherlands) or rabbit anti-C5b-9 (5 µg/mL, a kind gift from Professor Paul Morgan, Cardiff University, UK) at 4°C overnight, or negative control rabbit IgG (Dako) at the same concentrations. Tissues were assessed in a blinded fashion. Fluorescence in each glomerulus was scored from 0 to 3 using a template presented in **Supplementary Figure 3**. Each score was multiplied by the number of glomeruli with that score in the entire kidney section. The sum of all fluorescence was calculated and divided by the total number of glomeruli in that section, to establish the mean fluorescence/glomerulus.

### Statistics

Statistical analysis was performed using GraphPad prism software (GraphPad Software, Version 9, La Jolla, Ca). The non-parametric Mann–Whitney U test was used for two group comparisons. Multiple group comparisons were performed using the Kruskal–Wallis test followed by Dunn’s multiple comparisons test. Cell experiments performed on the same day with the same serum donor, stimulated with and without kaolin or FXIIa and in the presence and absence of R715 or 2-APB, were not considered independent, and thus data were analyzed as paired using the non-parametric Wilcoxon signed rank test. A P value ≤0.05 was considered significant.

## Results

### B1 receptor on glomerular endothelial cells triggers complement activation

**Figure 2:**
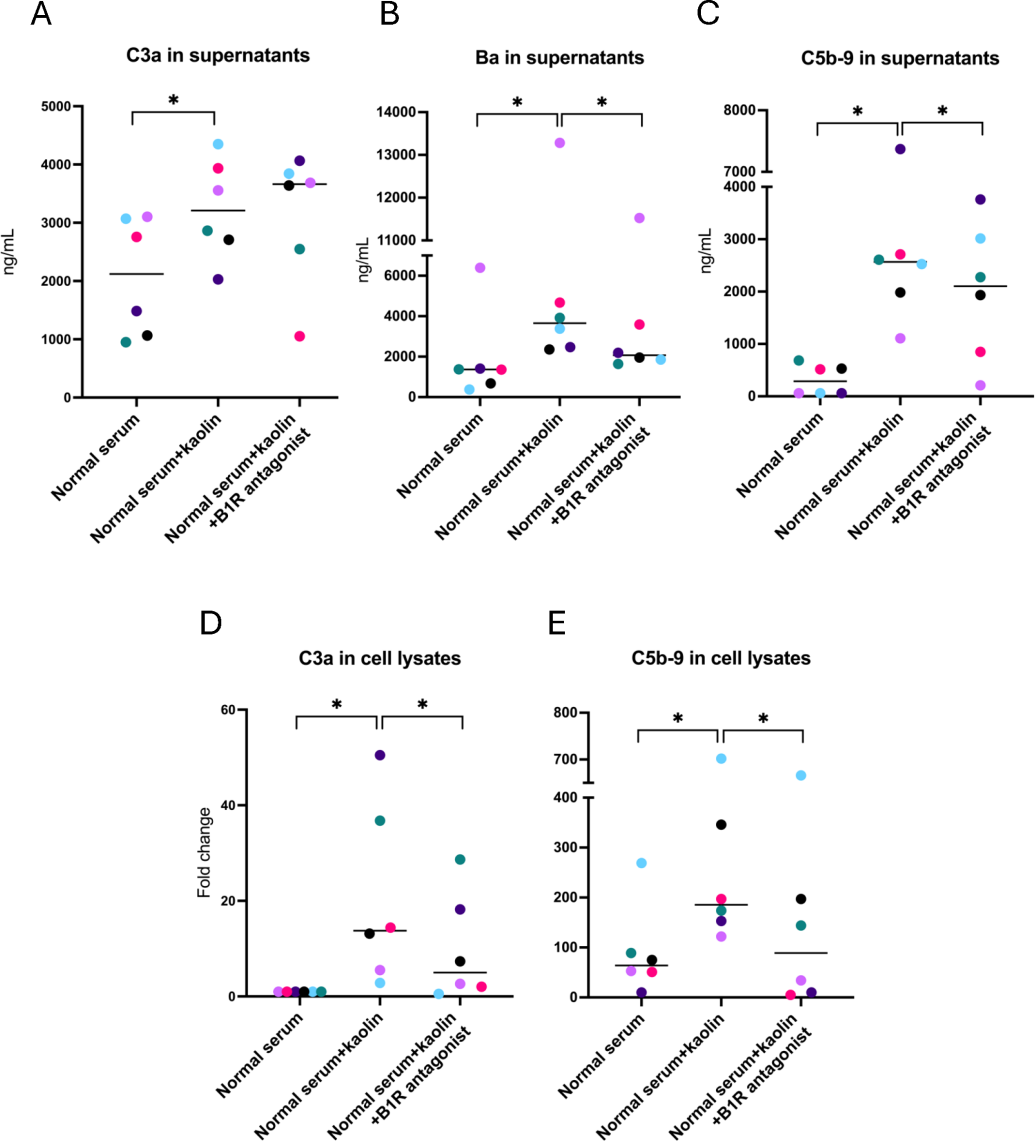
Complement activation in glomerular endothelial cells incubated with kaolin and the effect of the B1R antagonist. **(A)** C3a in glomerular endothelial cell supernatants. **(B)** Factor Ba in glomerular endothelial cell supernatants. **(C)** C5b-9 in glomerular endothelial cell supernatants. **(D)** C3a in glomerular endothelial cell lysates. Differences in C3a staining are presented as fold change, in which cells incubated with normal serum alone (left lane) were defined as 1. **(E)** C5b-9 in glomerular endothelial cell lysates. The Wilcoxon signed rank test for paired samples was used in which each color represents one experiment. *P < 0.05. B1R, bradykinin receptor 1.

Supernatants from PGECs incubated with kaolin or FXIIa, in the presence or absence of the B1R-antagonist R715, were analyzed for C3a, Ba, and C5b-9. C3a in supernatants was increased when cells were activated with kaolin, and the B1R antagonist did not lower concentrations of C3a (**Figure 2A**). Levels of Ba and C5b9 were elevated in supernatants incubated with kaolin (**Figures 2B, C**) and significantly reduced in the presence of the B1R-antagonist. Cell lysates activated with kaolin showed higher levels of both C3a (**Figure 2D**) and C5b-9 (**Figure 2E**), and these levels were significantly reduced in the presence of the B1R-antagonist. Levels of Ba were not assayed in cell lysates because Ba is not cell-bound.

**Figure 3:**
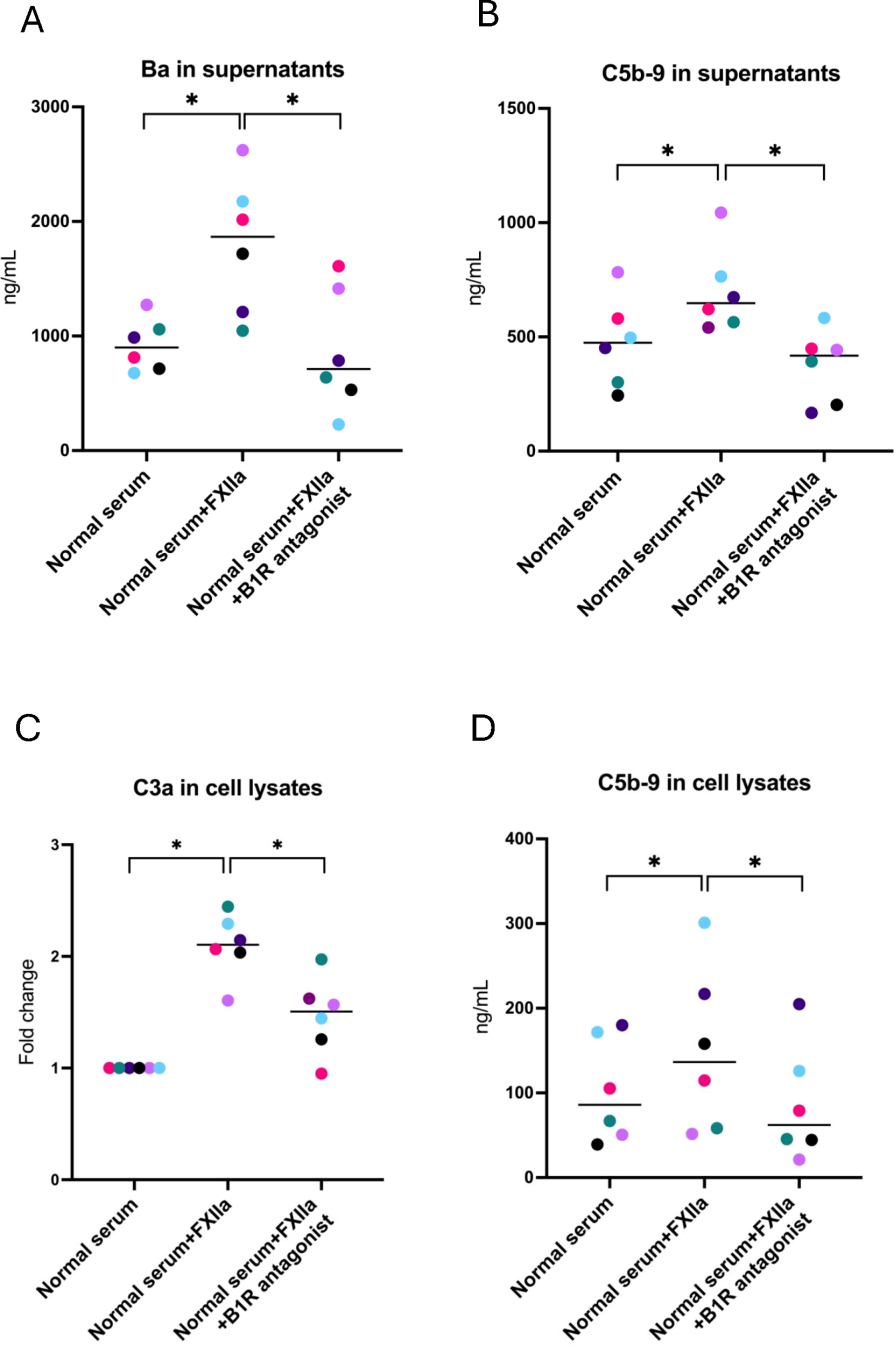
Complement activation in glomerular endothelial cells incubated with FXIIa, and the effect of the B1R antagonist. **(A)** Factor Ba in glomerular endothelial cell supernatants. **(B)** C5b-9 in glomerular endothelial cell supernatants. **(C)** C3a in glomerular endothelial cell lysates. Differences in C3a staining are presented as fold change, in which cells incubated with normal serum alone (left lane) were defined as 1. **(D)** C5b-9 in glomerular endothelial cell lysates. The Wilcoxon signed rank test for paired samples was used, in which each color represents one experiment. *P < 0.05. B1R, bradykinin receptor 1.

Supernatants incubated with FXIIa were likewise analyzed for C3a, Ba, and C5b-9. Concentrations of Ba (**Figure 3A**) and C5b-9 (**Figure 3B**) were elevated when incubated with FXIIa and significantly lowered in the presence of the B1R antagonist. Levels of C3a in supernatants were not elevated when activated with FXIIa, and no reduction was seen in the presence of the B1R antagonist (**Supplementary Figure 4**). Cell lysates showed higher levels of C3a (**Figure 3C**) and C5b-9 (**Figure 3D**) in the presence of FXIIa, which were significantly reduced in the presence of the B1R antagonist.

Control experiments demonstrated that FXIIa did not cleave C3 to C3a (**Supplementary Figure 5A**) or affect levels of C5b-9 in a pure system (**Supplementary Figure 5B**). In addition, normal human serum incubated with kaolin or FXIIa in the absence of cells did not generate C3a (**Supplementary Figure 5C**, zymosan was used as the positive control).

### Kinin receptor-mediated complement activation was reduced by an inositol 1,4,5-trisphosphate receptor antagonist

**Figure 4:**
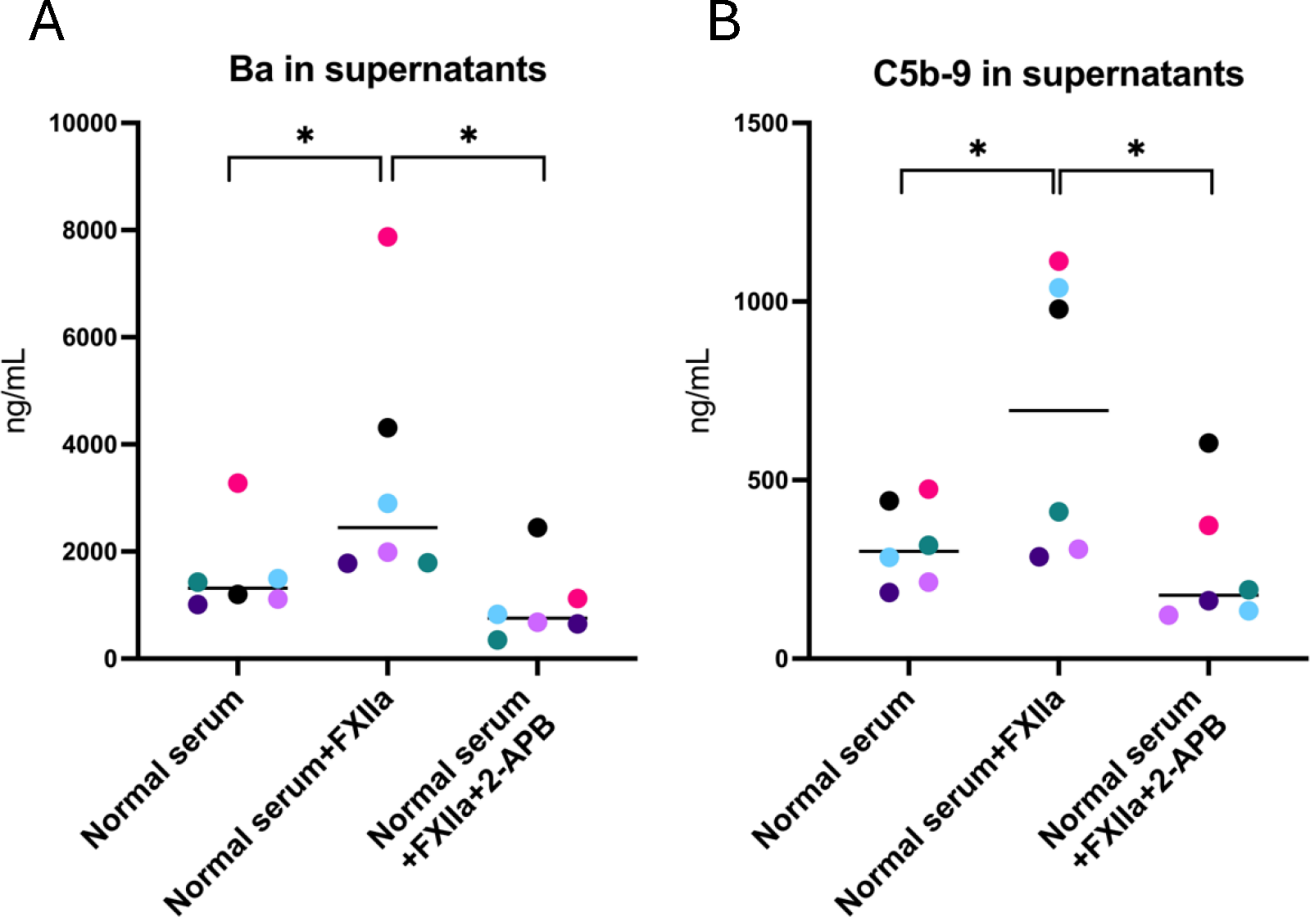
Complement activation of glomerular endothelial cells stimulated with FXIIa and the effect of IP3-receptor inhibition. **(A)** Factor Ba in glomerular endothelial cell supernatants. **(B)** C5b-9 in glomerular endothelial cell supernatants. Wilcoxon’s signed rank test was used for statistical comparisons and experiments are sorted by color. *P < 0.05. 2-APB, 2-aminoethoxydiphenyl borate.

2-APB is an IP3 receptor antagonist. The following experiments were performed to investigate if 2-APB affected FXIIa-induced complement activation, by blocking one of the intracellular signaling pathways of GPCRs. Supernatants and cell lysates from PGECs incubated with FXIIa in the presence or absence of 2-APB were analyzed for complement activation. Ba (**Figure 4A**) and C5b-9 (**Figure 4B**) were increased in supernatants of cells incubated with FXIIa and lowered in the presence of 2-APB. C3a levels in cell supernatants were not tested in the presence of 2-APB because these were not elevated by incubation with FXIIa, as described above. The effect of 2-APB on cell lysates could not be tested as the DMSO vehicle in itself decreased complement levels in lysates but did not affect complement measurements in supernatants. 2-APB did not affect kallikrein activity (**Supplementary Figure 1C**).

### LPS injection in mice decreased prekallikrein

**Figure 5:**
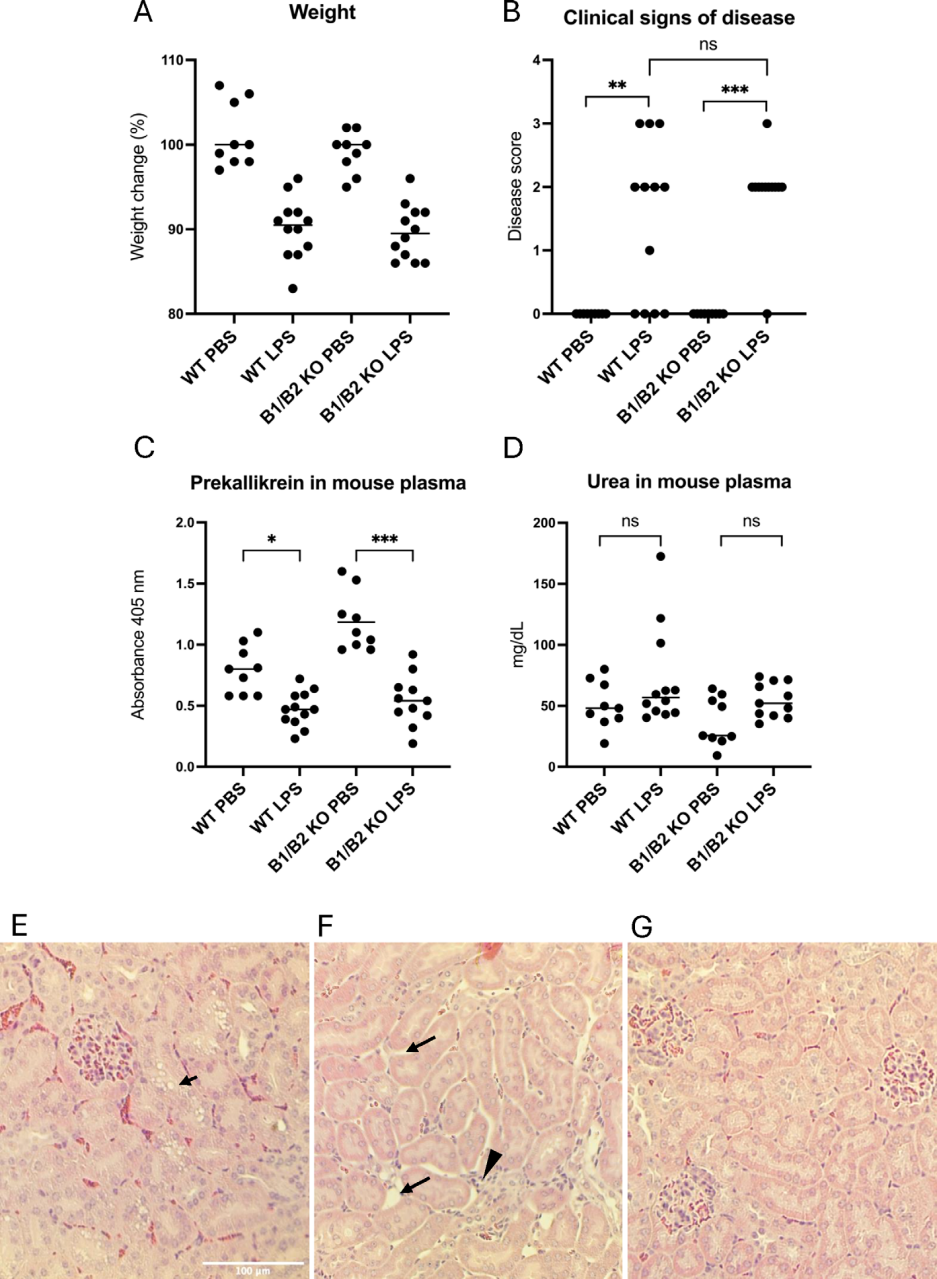
Wild-type and B1/B2 receptor knockout mice injected with LPS. **(A)** Body weight changes in mice treated with LPS (wild-type n=12, B1/B2 receptor knockout n=12) or PBS (wild-type n=9, B1/B2 receptor knockout n=9). Each dot represents one mouse, and the bar represents the median. **(B)** Clinical signs of disease scored in the same mice. A score of 0–3 was given to each mouse at the end of the experiment, as per **Supplementary Table 1**. **(C)** Prekallikrein in plasma samples from the same mice. **(D)** Plasma urea concentrations of the mice. **(E)** A representative kidney section from an LPS-treated wild-type mouse. Arrow indicates epithelial vacuolization. **(F)** A representative kidney section from an LPS-treated wild-type mouse. Arrow indicates interstitial widening, suggesting edema. Arrowhead indicates infiltrative cells. **(G)** A representative kidney section from a wild-type mouse given PBS, showing normal mouse kidney histology. Kruskal–Wallis test followed by Dunn’s multiple comparison used for statistical comparisons in panels A-C. *P<0.05, **P< 0.01, ***P<0.001, ns: not significant. LPS, lipopolysaccharide, WT, wild-type, B1/B2, B1/B2 receptor, KO, knockout.

B1/B2 receptor knockout mice (n=21) and wild-type mice (n=21) were treated with LPS to induce kallikrein–kinin system activation or injected with the PBS vehicle, 12 treated mice and 9 controls in each group. Mice were sacrificed within 14 h–24 h. All mice injected with *E. coli* O55:B5 LPS exhibited weight loss compared with controls (**Figure 5A**), and most of them exhibited clinical signs of disease, regardless of genotype (**Figure 5B**; for the disease score, see **Supplementary Table 1**). Prekallikrein was consumed in the plasma of LPS-treated mice (**Figure 5C**), indicating kallikrein activation. Urea levels were slightly higher in LPS-treated mice versus untreated mice, but this did not achieve statistical significance (**Figure 5D**).

### LPS-treated mice exhibited histopathological changes in the kidneys

LPS-injected mice exhibited histopathological changes in kidney sections not present in PBS-control mice (**Table 1**). The changes observed were vacuolization of tubular cells (**Figure 5E**) and interstitial widening suggestive of interstitial edema, as well as infiltrates (**Figure 5F**). Control mice exhibited no kidney pathology (**Figure 5G**). Pathology was more pronounced in mice taken after 24 h compared with 14 h. LPS-treated wild-type mice exhibited significantly more epithelial desquamation, compared with LPS-treated B1/B2 receptor knockout mice. Edema and epithelial vacuolization were slightly more prominent in LPS-treated wild-type mice compared with B1/B2 receptor knockout mice, but this did not achieve statistical significance (**Table 1**). No pathological changes were seen in glomeruli.

**Table 1:** Kidney histopathology in LPS-treated mice.

Histopathological finding	LPS-treated (n=14)		Controls (n=6)	
	WT (n=7)	B1R/B2R KO (n=7)	WT (n=3)	B1R/B2R KO (n=3)
Tubular vacuolization	1 (1-3)^a^	1 (0-2)	0	0
Tubular epithelial desquamation	1 (0-2)*	0 (0-1)	0	0
Interstitial edema	1 (0-2)	1 (0-1)	0	0

a Scores are presented as median; range is presented in parentheses. An entire kidney section was analyzed from each mouse. Histopathological changes were scored in a blinded fashion as 0 (absent), 1 (mild), 2 (moderate), and 3 (severe). Statistical comparisons were performed using Kruskal–Wallis test followed by Dunn’s multiple comparison, comparing LPS-treated wild-type to LPS-treated B1/B2 receptor knockout mice. *P < 0.05. LPS, lipopolysaccharide; WT, wild type; B1R/B2R, B1/B2 receptor; KO, knockout.

### Mice treated with LPS displayed complement deposition in kidneys

**Figure 6:**
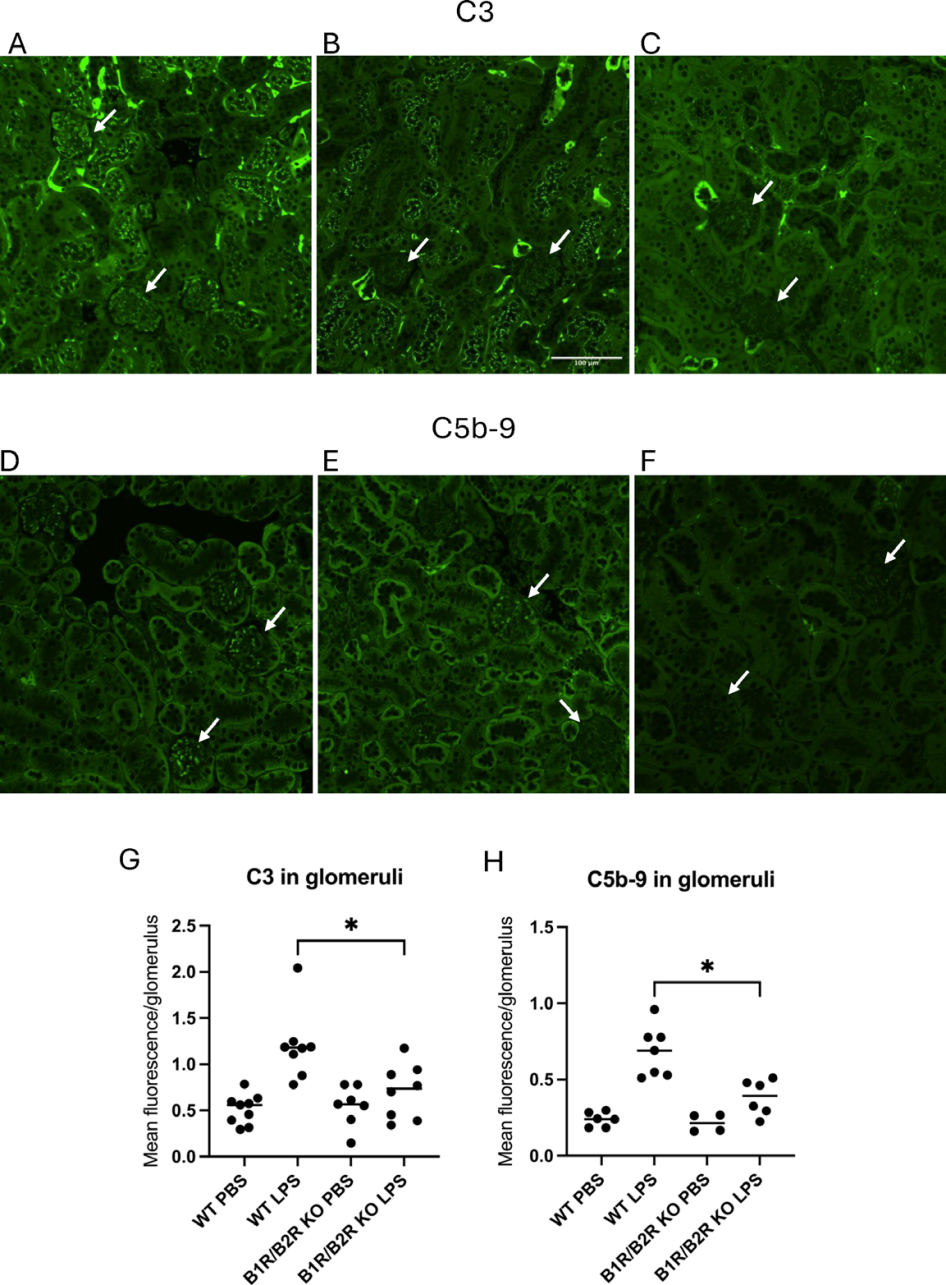
C3 and C5b-9 in kidneys in LPS-treated wild-type and B1/B2 receptor knock out mice. **(A)** C3 in a representative kidney section from an LPS-treated wild-type mouse. Arrows indicate glomeruli scored as 1 (lower glomerulus) and 3 (upper), respectively. **(B)** C3 staining in a representative kidney section from an LPS-treated B1/B2 receptor knockout mouse. Arrows indicate two glomeruli scored as 0. **(C)** C3 staining of a representative kidney section from a PBS vehicle-treated wild-type mouse exhibiting mild staining in tubules but not in glomeruli. Arrows indicate two glomeruli scored as 0. **(D)** C5b-9 in a representative kidney section from an LPS-treated wild-type mouse. Arrows indicate glomeruli scored as 1 (upper) and 2 (lower), respectively. **(E)** C5b-9 in a representative kidney section from an LPS-treated B1/B2 receptor knockout mouse. Arrows indicate glomeruli scored as 0 (lower) and 1 (upper), respectively. **(F)** C5b-9 in a representative kidney section from a PBS vehicle-treated wild-type mouse injected with PBS stained for C5b-9. Arrows indicate two glomeruli scored as 0. **(G)** Kidney sections from wild-type mice injected with PBS (n=9), LPS (n=8), B1/B2 receptor knockout mice injected with PBS (n=7), or LPS (n=8) were stained for C3, and the intensity was calculated and presented here as mean fluorescence/glomerulus. **(H)** Kidney sections from wild-type mice injected with PBS (n=6) or LPS (n=7) and B1/B2 receptor knockout mice injected with PBS (n=4) or LPS (n=6) and stained for C5b-9; the intensity was calculated and presented here as mean fluorescence/glomerulus. Kruskal–Wallis test followed by Dunn’s multiple comparison was used for statistical comparisons. *P<0.05. LPS, lipopolysaccharide; WT, wild type; B1/B2, B1/B2 receptor; KO, knockout.

Kidney sections from LPS-treated mice were stained for C3 and C5b-9 and compared with mice treated with the PBS vehicle. LPS-treated wild-type mice exhibited significantly more C3 staining in glomeruli (**Figure 6A**) compared with the B1/B2 receptor knockout mice (**Figure 6B**) and PBS controls. The latter showed minimal glomerular staining (**Figure 6C**). Likewise, LPS-treated wild-type mice showed more C5b-9 staining (**Figure 6D**) than B1/B2 receptor knockout mice (**Figure 6E**) and controls (**Figure 6F**). Complement deposition in glomeruli was scored in most mice, and fluorescence levels/glomerulus are presented for C3 in **Figure 6G** and for C5b-9 in **Figure 6H**, showing that LPS-treated wild-type mice had significantly more complement deposition than B1/B2 receptor knockout mice whereas the PBS controls had minimal glomerular complement staining.

## Discussion

FXIIa stimulates kallikrein activation leading to HK cleavage and the release of bradykinin and DABK, which bind the two kinin receptors B2R and B1R, respectively. Here, we show that kaolin and FXIIa induce complement activation on the glomerular endothelium in a B1R-dependent manner. Complement activation was detected by an increase in C3a and C5b-9 in cell supernatants and lysates. Increased C5b-9 suggests that even the terminal complement pathway was activated potentially leading to endothelial cell injury. Additionally, increases of the factor B cleavage product, Ba, in cell supernatants suggests that FXIIa-induced complement activation occurs via the alternative pathway. Complement release and deposition was, at least in part, triggered by B1R signaling as the B1R antagonist as well as the IP3R antagonist, which blocks the intracellular signaling pathway of GPCRs, both reduced complement activation. In wild-type mice, LPS stimulation led to kallikrein activation, as well as complement deposition in glomeruli. Although kallikrein activation was similar, complement activation was reduced in B1R/B2R double knockout mice. Taken together, the *in vitro* and *in vivo* data suggest that signaling via bradykinin receptors, and particularly B1R, contributes to complement activation on the glomerular endothelium. As complement C3a is an anaphylatoxin and C5b-9 is associated with cell activation or membrane injury, the presence of these complement proteins on the cell could contribute to vascular inflammation.

LPS can induce complement activation (34) particularly by activating the alternative pathway (35). In the current study, LPS was used to stimulate kallikrein activity, as bacterial endotoxins have been shown to activate FXII *in vitro* (36) and induce the KKS *in vivo* (37). As LPS can induce activation of both complement and the KKS, we cannot rule out that C3 and C5b-9 deposition in glomeruli of wild-type mice was due to direct activation of the alternative pathway of complement. However, the B1R/B2 receptor double-knockout mice exhibited significantly less C3 and C5b-9 deposition, indicating that complement deposition was partly associated with signaling via these kinin receptors.

Kallikrein was previously demonstrated to cleave C3 (23). Thus, kaolin or FXIIa could activate kallikrein and induce complement activation extracellularly. The inhibitory effects of the B1R antagonist R715 and the IP3R inhibitor 2-APB suggest, however, that intracellular activation is occurring as well. Bradykinin receptor signaling occurs by activation of phospholipase C and IP3-associated release of calcium into the cytoplasm (38). By blocking the B1R and IP3R *in vitro*, and by studying the effects of LPS in B1R/B2R knockout mice, we show that complement deposition is associated with cellular signaling via kinin receptors.

We have previously demonstrated that vasculitis plasma perfused over glomerular endothelial cells led to release of complement-coated endothelial extracellular vesicles. B1R and B2R antagonists reduced complement deposition on the vesicles (26). Complement deposition can be harmful for cellular integrity, particularly formation of C5b-9 in the membrane; thus, the cell may rid itself of harmful substances by releasing extracellular vesicles. Here, we show that complement is also deposited on the glomerular endothelial cells themselves, in response to activation of the KKS. Thus, the cells cannot rid themselves entirely of complement by shedding vesicles and blocking the kinin receptors may further reduce complement deposition.

Endothelial cells were stimulated with kaolin or FXIIa, and the B1R antagonist decreased complement C3a and C5b-9 deposition on cell lysates. The B1R antagonist did not, however, reduce C3a in supernatants, most probably because the effect is most prominent on cell membranes when C3a is bound to its receptor. We previously showed that a B1R antagonist decreased glomerular C3 deposition in a glomerulonephritis mouse model (26), which is in line with the results presented herein, showing the importance of both the kinin receptors for mediating complement activation in glomeruli *in vivo*. These results collectively suggest that inhibitors of the KKS and its receptor signaling can decrease complement activation on the glomerular endothelium during vascular inflammation.

Conditions with profound vascular inflammation in the kidney may potentially benefit from KKS inhibition. For example, patients with diabetes nephropathy have complement deposits in the kidney (39) and are often treated with angiotensin-converting enzyme (ACE) inhibitors to reduce proteinuria. ACE inhibitors increase bradykinin concentrations by blocking its degradation (40). Thus, conditions in which both complement and kinin proteins or peptides may negatively affect the kidney could benefit from blockade of kinin receptors.

There are currently several approved pharmaceutical agents that can effectively block activation of the KKS, and more are under development. Most of them are used as treatment or prophylaxis in hereditary angioedema. These treatments include the C1 inhibitor, a monoclonal antibody targeting FXII, garadacimab, and kallikrein inhibitors (41). All these inhibitors will inhibit the generation of kinin peptides, and hence their signaling through B1R and B2R. Icatibant is the only approved kinin receptor antagonist, targeting the B2R (42). To date, there is no commercially available inhibitor of the B1R.

The B1R is induced by inflammatory stimuli, and upon ligand binding, it does not desensitize and thus generates prolonged intracellular signaling (16). Therefore, the B1R is of importance during chronic inflammation. In a mouse model of late-stage sepsis, treatment with a B1R antagonist improved survival rates and reduced kidney injury and lung lesions (4). The results of the current study suggest that KKS inhibition by a B1R antagonist may have a beneficial effect in vascular inflammation, which should be addressed in clinical trials.

## Data Availability

The original contributions presented in the study are included in the article/**Supplementary Material**, further inquiries can be directed to the corresponding author/s.
