# Supplementary material for: Bradykinin B1 receptor signaling triggers complement activation on endothelial cells
Source: Front Immunol. 2025 Feb 7;16:1527065. doi: 10.3389/fimmu.2025.1527065 (PMC11842366; doi:10.3389/fimmu.2025.1527065)
Supplement: Supplementary file 1 [file DataSheet1.pdf]

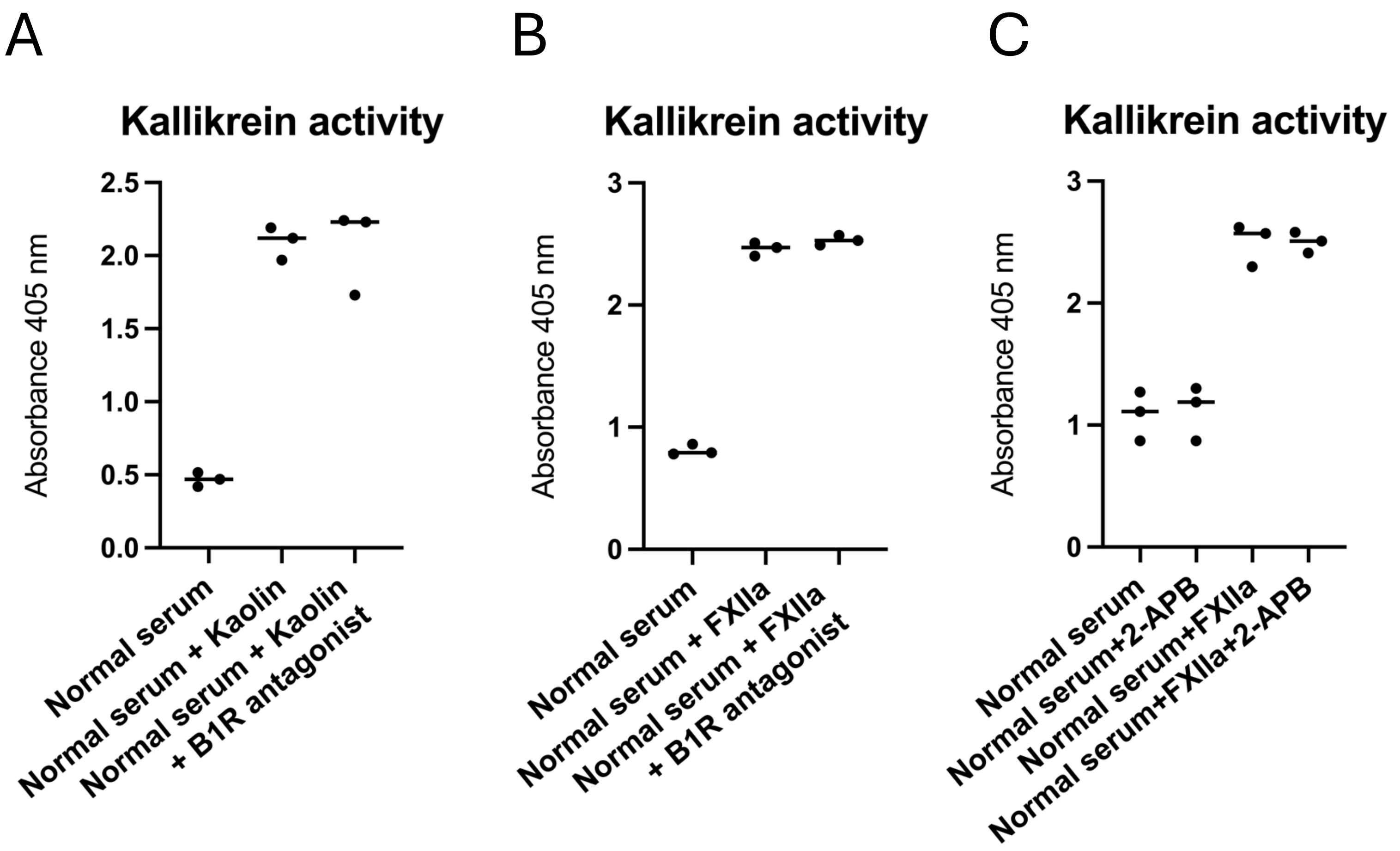

Supplementary Figure 1: Activation of the kallikrein kinin system in PGEC supernatants was not affected by the B1R antagonist or 2-APB A) Kallikrein activity in supernatants from cells stimulated with kaolin with and without the B1R antagonist. **B)** Kallikrein activity in supernatants from cells stimulated with FXIIa with and without the B1R antagonist. **C)** Kallikrein activity in supernatants from cells incubated with FXIIa with and without 2-APB. B1R: Bradykinin 1 receptor, 2-APB: 2-Aminoethoxydiphenyl borate, FXIIa: factor XIIa

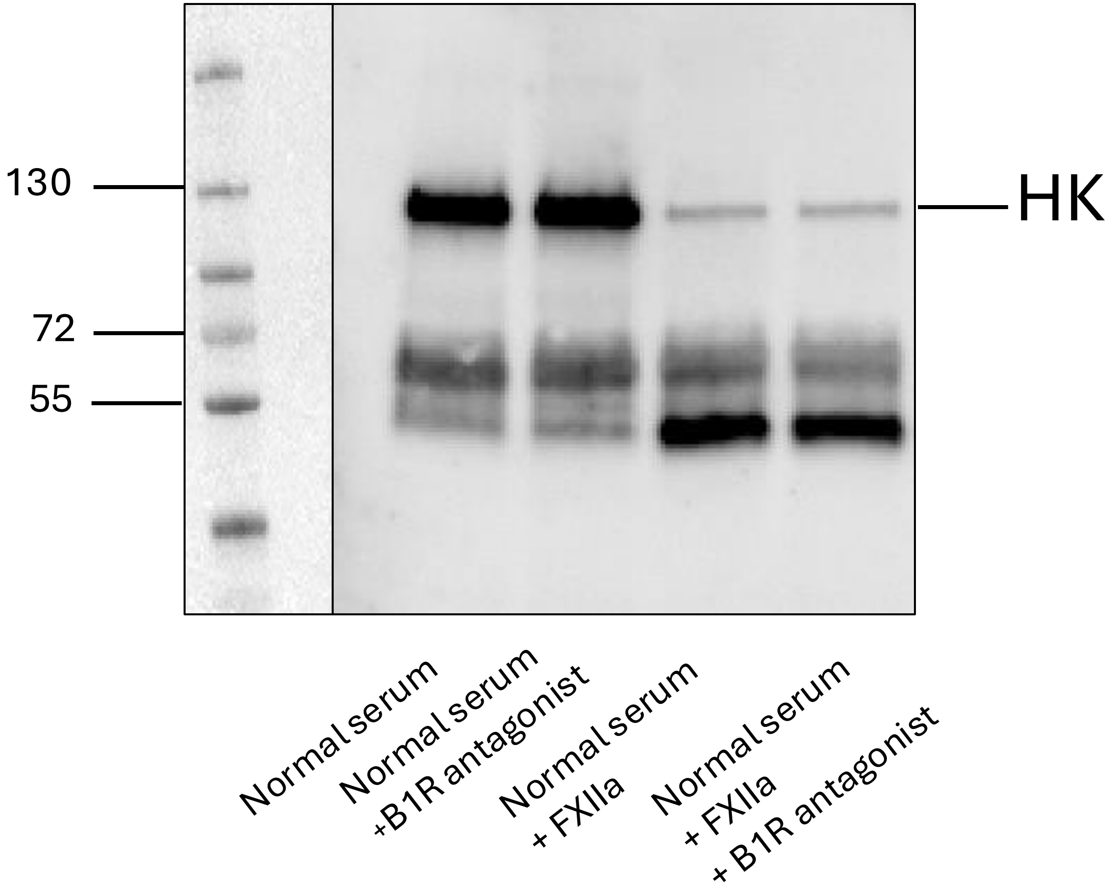

Supplementary Figure 2: Immunoblot of high molecular weight kininogen (HK) and its degradation products in supernatants from PGECs incubated with or without factor XIIa (FXIIa) and in the absence and presence of the bradykinin receptor 1 (B1R) antagonist.

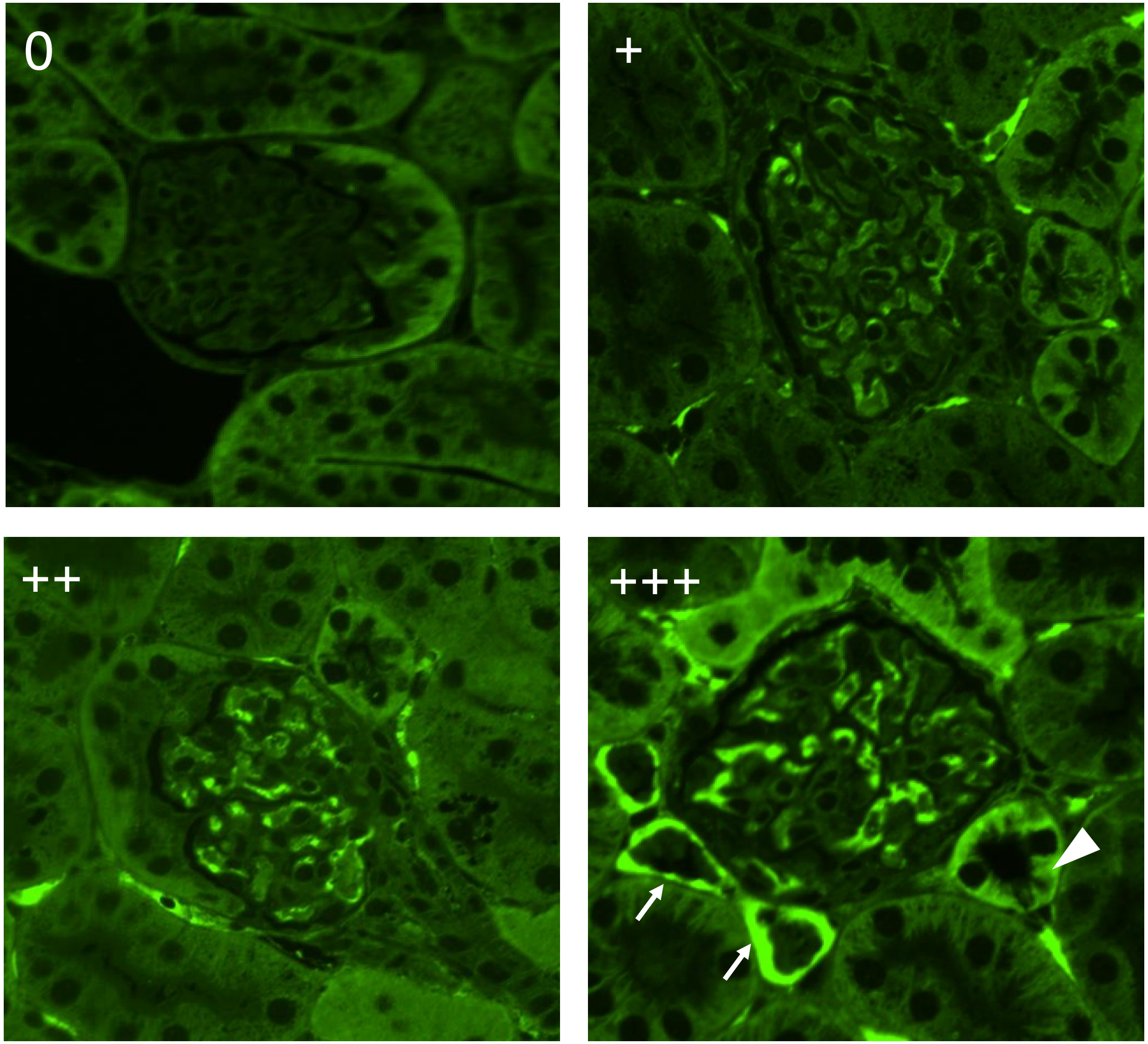

Supplementary Figure 3: Kidney sections from mice injected with LPS or PBS, stained for C3 or C5b-9 graded after fluorescent intensity. Each glomerulus in a section was counted and given a score using a scoring system of no staining (0), low (+), medium (++) and high intensity (+++). This figure was used as a template when grading sections. Only staining of glomeruli was used for scoring. Arrows indicate positive staining in arerioles. Arrowheads show staining in tubular cells.

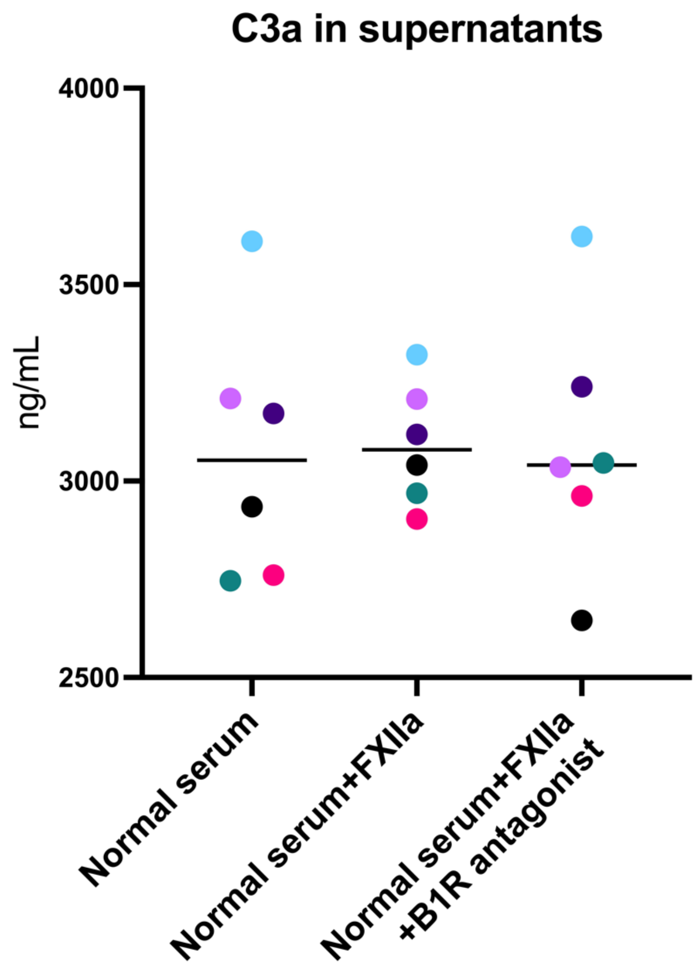

Supplementary Figure 4: C3a in the supernatant of primary glomerular endothelial cells incubated with FXIIa. C3a in supernatants from cells stimulated with FXIIa, with and without the bradykinin receptor 1 antagonist. Each color represents one experiment. B1R: Bradykinin receptor 1, FXIIa: activated factor XII.

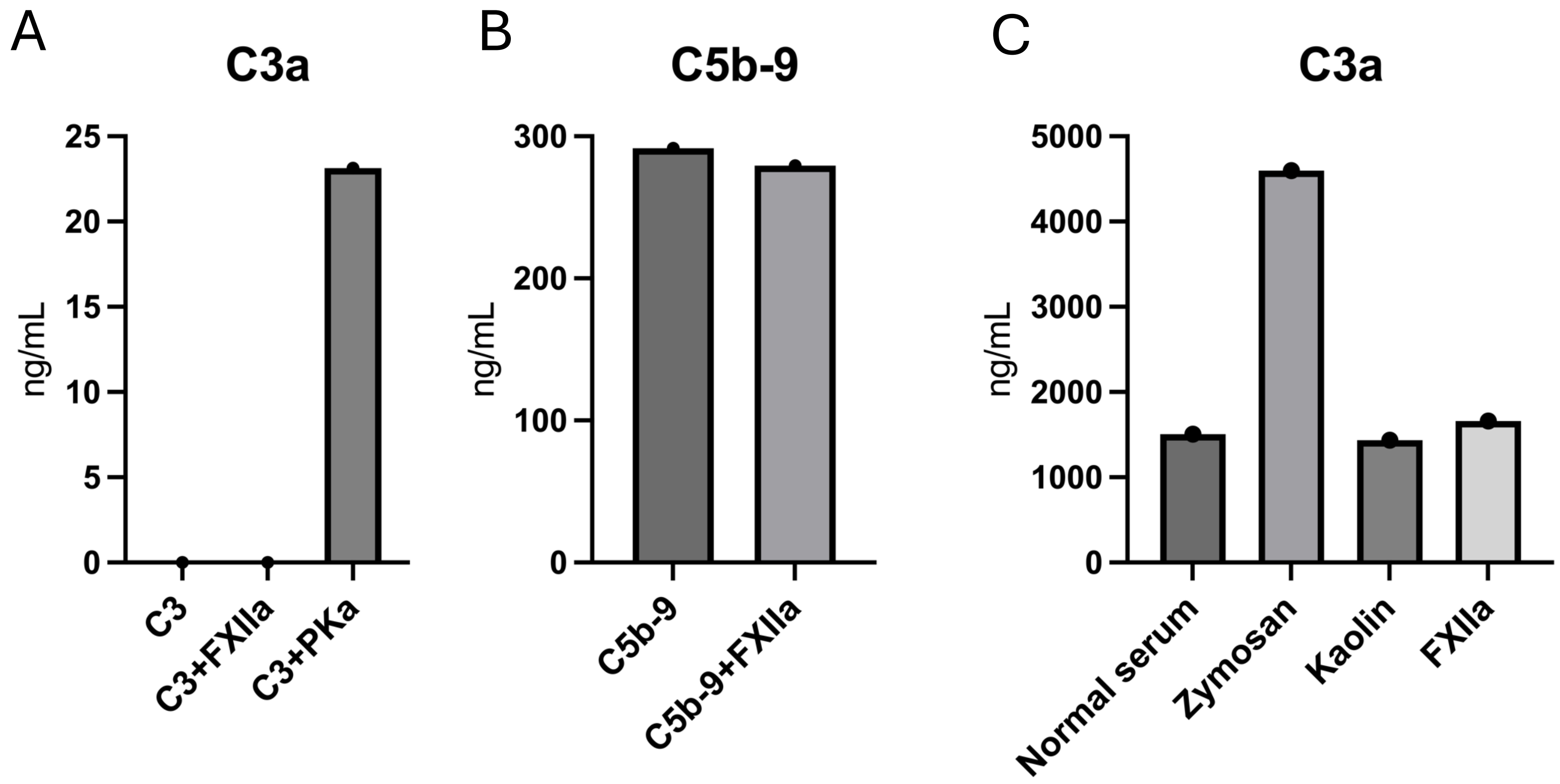

Supplementary Figure 5: Complement activation by kaolin or FXIIa in the absence of cells. A) C3 incubated with FXIIa or kallikrein (PKa, positive control) in the absence of cells and serum, analyzed for C3a. **B)** C5b-9 incubated with FXIIa without serum or cells. **C)** C3a levels in normal serum incubated with zymosan (positive control), kaolin, or FXIIa in the absence of cells. These were control experiments.

Supplementary Table 1: Disease score

**Score**	**Characteristics**	**Clinical signs of disease**
**0**	None	-
**1**	Mild	Systemic: Ruffled fur<br>Neurologic: None
**2**	Moderate	Systemic: Ruffled fur, hunched posture, decreased activity, squinting<br>Neurologic: None
**3**	Severe	Systemic: Ruffled fur, hunched posture, severely decreased mobility, squinting, lethargy<br>Neurologic: Ataxia, rigidity
